# Predator-Induced Demographic Shifts in Coral Reef Fish Assemblages

**DOI:** 10.1371/journal.pone.0021062

**Published:** 2011-06-16

**Authors:** Benjamin I. Ruttenberg, Scott L. Hamilton, Sheila M. Walsh, Mary K. Donovan, Alan Friedlander, Edward DeMartini, Enric Sala, Stuart A. Sandin

**Affiliations:** 1 Marine Science Institute, University of California Santa Barbara, Santa Barbara, California, United States of America; 2 National Marine Fisheries Service, Southeast Fisheries Science Center, Miami, Florida, United States of America; 3 Moss Landing Marine Laboratories, Moss Landing, California, United States of America; 4 Scripps Institution of Oceanography, La Jolla, California, United States of America; 5 United States Geological Survey, Hawaii Cooperative Fishery Research Unit, University of Hawaii, Honolulu, Hawaii, United States of America; 6 National Marine Fisheries Service, Pacific Islands Fisheries Science Center, Aiea, Hawaii, United States of America; 7 Centre d'Estudis Avançats de Blanes, Consejo Superior de Investigaciones Científicas, Blanes, Spain; 8 National Geographic Society, Washington, D.C., United States of America; California Academy of Sciences, United States of America

## Abstract

In recent years, it has become apparent that human impacts have altered community structure in coastal and marine ecosystems worldwide. Of these, fishing is one of the most pervasive, and a growing body of work suggests that fishing can have strong effects on the ecology of target species, especially top predators. However, the effects of removing top predators on lower trophic groups of prey fishes are less clear, particularly in highly diverse and trophically complex coral reef ecosystems. We examined patterns of abundance, size structure, and age-based demography through surveys and collection-based studies of five fish species from a variety of trophic levels at Kiritimati and Palmyra, two nearby atolls in the Northern Line Islands. These islands have similar biogeography and oceanography, and yet Kiritimati has ∼10,000 people with extensive local fishing while Palmyra is a US National Wildlife Refuge with no permanent human population, no fishing, and an intact predator fauna. Surveys indicated that top predators were relatively larger and more abundant at unfished Palmyra, while prey functional groups were relatively smaller but showed no clear trends in abundance as would be expected from classic trophic cascades. Through detailed analyses of focal species, we found that size and longevity of a top predator were lower at fished Kiritimati than at unfished Palmyra. Demographic patterns also shifted dramatically for 4 of 5 fish species in lower trophic groups, opposite in direction to the top predator, including decreases in average size and longevity at Palmyra relative to Kiritimati. Overall, these results suggest that fishing may alter community structure in complex and non-intuitive ways, and that indirect demographic effects should be considered more broadly in ecosystem-based management.

## Introduction

Understanding the factors that lead to variation in coral reef fish populations and community structure is critically important to improving conservation and fisheries management. Over the past decades, it has become increasingly clear that human activities have altered ecological structure in many coastal systems through processes such as fishing, pollution, and climate change [1]–[3]. Fishing has a variety of direct effects on most coral reef ecosystems, and commercial, recreational, and subsistence reef fish fisheries exist throughout the tropics. Changes in individual sizes of target species and subsequent alterations of community structure resulting from fishing have been well-documented in many systems worldwide [1], [2], [4], [5]. However, these effects are likely not restricted to changes in abundance or size, but may include changes in demography and life histories that are more difficult to detect, and yet may still strongly influence the ecology of these systems.

Few studies have examined changes in demography and life history of individual species as a result of fishing, and nearly all of these have examined fishery target species [6]–[10]. Findings commonly show that fishing directly reduces longevity, mean and maximum size, and the size and age at maturation and sex change of targeted fishery species. A number of studies have examined spatial variability in demography and life-history of non-target reef fish, but these studies have generally focused on the influence of large-scale geography or environmental factors such as temperature [11]–[16]. Indirect effects of fishing on demographic patterns of non-target species have been generally neglected (but see [17], [18]), in part because detecting such changes can be difficult, especially in systems that have experienced significant changes in community structure as a result of overexploitation.

Indirect effects can result from cascading effects of removal (or restoration) of top trophic groups. In most coral reef systems, top predators (e.g., sharks and larger groupers, snappers, and jacks) are often the most sought-after species in a fishery and therefore subject to the strongest fishing pressure. General ecological theory predicts that reductions in abundances of top predators should lead to reductions in rates of predation on lower trophic groups and ‘prey release,’ i.e. the increase in prey populations following reductions of predator density [4], [19], which may result in a classic trophic cascade. Trophic cascades are common in many ecosystems, but are less prevalent in marine environments. When present, trophic cascades often involve sea urchins or other echinoderms and specialist predators, and are generally more common in temperate than tropical regions [20]–[22]. The complexity of tropical marine food webs appears to reduce the prevalence of prominent trophic cascades, and there are few if any documented trophic cascades in coral reef fish assemblages ([19] but see [23], [24]). Even though numerical trophic cascades are rare in marine systems, indirect effects on demography or life histories of prey species may occur, and such indirect effects may have strong impacts throughout the community. For example, changes in species composition, size or longevity of grazers can strongly influence grazing rates, which can in turn influence abundance of some groups of algae that compete with corals for space on the reef [25]–[27].

Because trophic connections among coral reef fish species are diffuse within complex food webs, we may not expect simple linear trophic cascades, in which decreases in top predators lead to increases in secondary consumers, resulting in decreases in primary consumers, and so forth. Descriptions of reef fish assemblages from the Caribbean [28], the central Pacific [29], and the Indian Ocean [30] show no clear evidence of guild-level release and commensurate trophic cascades within fish assemblages, even among areas that differ in abundance of top predators. Instead, top predators on coral reefs tend to be generalists and are likely feeding on a variety of fishes and invertebrates from several lower trophic groups.

Although there is little evidence that fishing results in prey release or trophic cascades on coral reefs, demographic traits of prey may still respond to removal of top predators. For example, when predatory fish are removed by fishing, demographic traits such as size and longevity may increase for non-targeted species in lower trophic levels because of reductions of predation intensity or predation risk. Therefore, we predict that where top predators are removed by fishing, prey fishes will live longer and attain larger sizes. If changes in predation and associated demographic shifts are extreme or persistent enough, we expect that changes in growth and life history may also occur for these species, analogous to those observed in directly exploited species [31].

In most coral reef locations, fish assemblages have been significantly altered for decades or even centuries due to anthropogenic influences, such as fishing, reductions in water quality, and loss of live coral and other associated habitats (e.g. seagrasses, mangroves). In many locations, densities of top predators, such as sharks and large snappers and groupers, have been so reduced that they no longer serve whatever ecological role they may have had in the past [2], [32]–[36]. However, the ideal system to examine predator-induced demographic shifts should include some areas that are free or nearly free of human and/or fishing impacts and other areas that are subject to fishing pressure. The Northern Line Islands in the central Pacific possesses many of these ideal characteristics. Recent work in this archipelago has found significant changes in population and community structure along a gradient of human disturbance, including changes in fish assemblages [18], benthic communities [29], microbial communities [37], and parasite communities [38]. Most importantly for examining indirect demographic shifts in coral reef fishes, top predator abundance in unfished locations is nearly as high as any other documented coral reef system, and is much lower in the fished areas [18], [29], [39]. We used two of the Northern Line Islands (unfished Palmyra and fished Kiritimati) to examine predator-induced demographic shifts in five different species of coral reef fishes from a variety of trophic levels. We found that top predators were larger and longer lived at unfished Palmyra relative to fished Kiritimati, but that species in lower trophic groups were generally smaller and experienced higher rates of mortality at Palmyra relative to Kiritimati. These results suggest that even the absence of clear numerical trophic cascades, demographic rates of non-target species may be influenced by the removal of top predators.

## Methods

**Figure 1:**
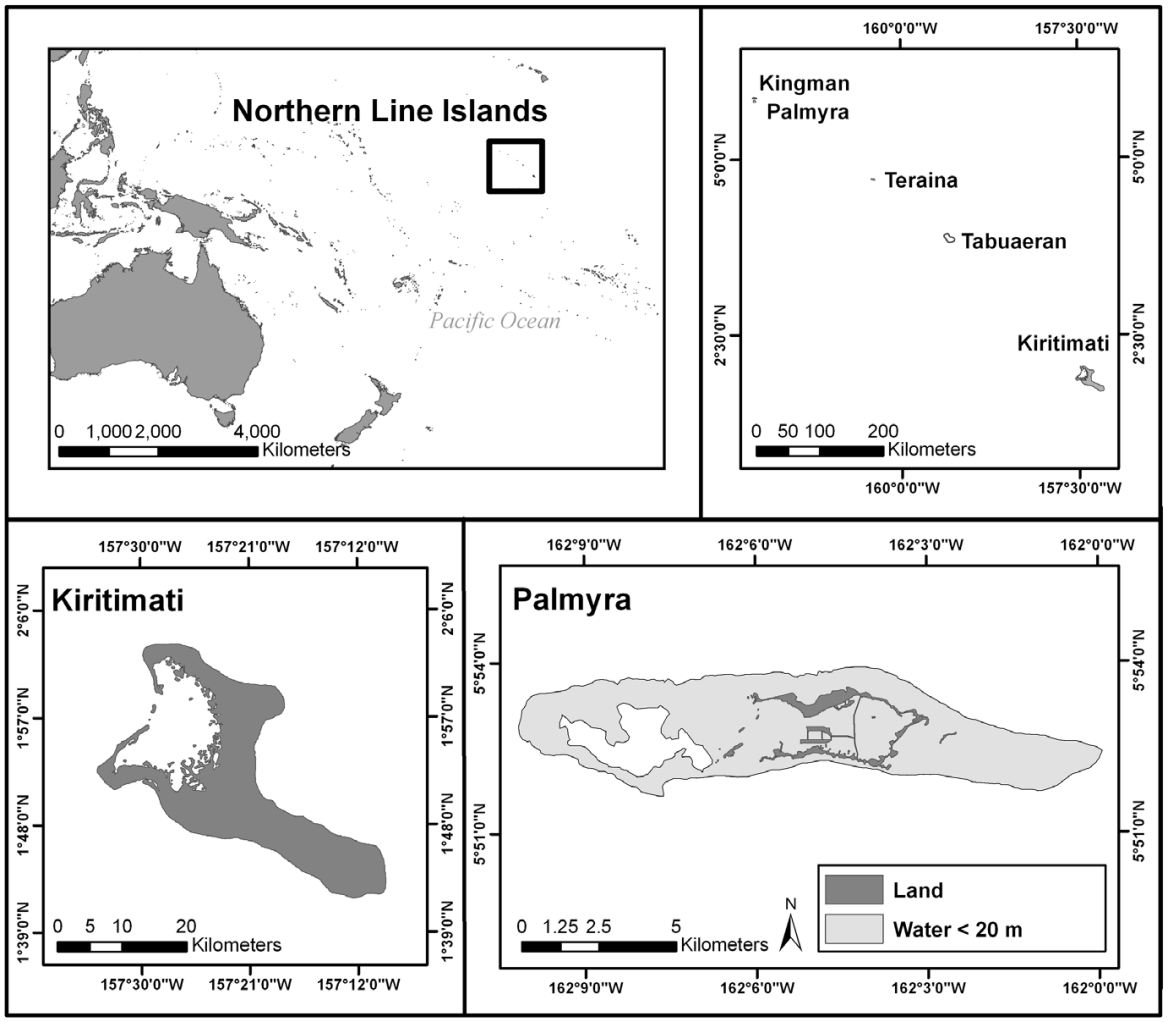
Map of the Line Islands, including Palmyra and Kiritimati.

The Northern Line Islands is a remote archipelago that spans 650 km between 1.75°–6.5°N and 157°–162.5°W in the central Pacific, located approximately 1500 km south of the Hawaiian Archipelago. Previous work in this archipelago has found that a number of putative metrics of ecosystem health, such as total fish biomass, predatory fish biomass, and cover of reef-builders (i.e. stony corals and crustose coralline algae) correlate negatively with human population size; conversely, putative metrics of ecosystem degradation, such as concentration of microbes, potential pathogens, and prevalence of coral disease correlate positively with human population [29], [37]. In this study, we compare fish assemblages between two of these islands with very different levels of human impacts, Palmyra Atoll and Kiritimati Atoll (Fig. 1). Palmyra has been a U.S. National Wildlife Refuge since 2001; fishing and non-scientific extraction are prohibited and were limited for decades prior, and the atoll houses only a small research station. Kiritimati Atoll, part of the Republic of Kiribati, has a population of ∼10,000 people, with most living on the northwestern part of the island where artisanal fishing and aquarium fish collecting are concentrated. The remote nature of the Line Islands makes conducting fieldwork difficult and expensive; we chose to sample Palmyra and Kiritimati because they differ greatly in human impacts and because they are the only two islands in the archipelago with managed runways, enabling reliable land-based field logistics.

Underwater visual surveys were used to quantify densities and size distributions for all non-cryptic species (see [18], [29] for methodological details). A pair of divers counted all fish within adjacent 25-m long transects, with the width of the transect scaled depending on the size of the fish (for each diver, a 4-m wide ‘swim-out’ counting all fish ≥20 cm total length [TL], and a 2-m wide ‘swim-back’ counting fish <20 cm TL). At each station, divers surveyed three transects, with the team surveying 600 m^2^ for larger fish and 300 m^2^ for smaller fish. Divers surveyed 26 stations at Palmyra and 25 stations at Kiritimati along the forereef at 10 m depth.

We selected among the most abundant species in each major trophic group for collections and detailed investigation of demography and life history. *Lutjanus bohar*, the two-spot red snapper, is a common top predator that feeds on fish and invertebrates and is preyed upon only by sharks. *Paracirrhites arcatus*, the arc-eye hawkfish, is a mid-level predator usually associated with small branching corals, which feeds primarily on benthic crustaceans. *Chromis margaritifer*, the bicolor chromis, is an abundant, shoaling planktivore. *Plectroglyphidodon dickii*, the blackbar devil, is an omnivore that establishes territories of algal turf but also feeds on small invertebrates. *Acanthurus nigricans*, the whitecheek surgeonfish, is a mobile herbivore that feeds primarily on turf algae. We used a variety of methods to collect individual fish, including netting, spearing, clove oil, hook and line, and assistance from local fishers on Kiritimati, attempting in all cases to obtain a representative sample of the size range observed in the field. All collections were made on the forereefs of the two islands, between 5 and 20 m depth. Because Palmyra is a National Wildlife Refuge, we were limited to collecting no more than 50 individuals of each species at that island and sample sizes were similar at Kiritimati. See Table 1 for a summary of collections.

**Table 1:** Samples sizes, mean numerical densities (abundance), parameter estimates for *L_inf_* and *k*, and ΔAIC values for each species at each island.

Species	Island	Sample Size (N)	Mean Density (# 100 m^−2^)	*L_inf_* (cm)	*k* (year^−1^)	ΔAIC
*L. bohar*	Kiritimati	65	1.5 (0.3)	30.4 (1.03)	0.3 (0.03)	−47.7
	Palmyra	20	4.1 (0.5)	49.9 (1.70)	0.1 (0.01)	
*P. arcatus*	Kiritimati	57	2.6 (0.4)	8.3 (0.20)	0.4 (0.03)	−9.7
	Palmyra	48	7.8 (0.9)	7.3 (0.23)	0.7 (0.08)	
*C. margaritifer*	Kiritimati	63	153.3 (25.6)	5.1 (0.12)	0.4 (0.03)	−11.2
	Palmyra	45	51.4 (7.9)	5.8 (0.14)	0.3 (0.02)	
*P. dickii*	Kiritimati	54	7.0 (1.5)	5.6 (0.12)	0.5 (0.05)	−4.0
	Palmyra	40	2.7 (0.5)	5.5 (0.11)	0.5 (0.04)	
*A. nigricans*	Kiritimati	67	1.9 (0.3)	15.3 (0.25)	0.7 (0.09)	−11.4
	Palmyra	42	7.0 (0.8)	14.3 (0.34)	0.5 (0.07)	

Values in parentheses are standard errors, and ΔAIC is the difference between AIC values for each species for separate VBGF models fit for each island compared to single model fit for each species.

Upon collection, samples were immediately transported to the lab for processing. Each specimen was weighed, measured, and sexed by gross examination of the gonads. The sagittal otoliths were removed, cleaned, and stored dry. Otolith preparation generally followed Robertson et al. [12]. Sagittal thin sections were examined under a compound microscope at 40–100× magnification using transmitted light. Annuli were interpreted using standard techniques [12] and each otolith was examined by at least two readers. When age estimates differed by more than 10%, a third reader examined the sample. When annuli were not interpretable after examination by three readers, the second sagitta was processed, and individuals for which both otoliths were unreadable were excluded from the analysis.

### Statistical Analyses

We calculated station-specific estimates of density and mean length for each species from survey data. To test for differences between islands, we compared these values using two-sample t-tests of station-specific estimates (*n* = 26 for Palmyra, *n* = 25 for Kiritimati), using log-transformations when necessary to reduce variance heterogeneity. To examine changes in size structure across all species in the assemblage, we calculated summary statistics of fish length estimates relative to species-specific maximum lengths. For each fish counted, we converted length to a proportion (between 0 and 1) reflecting the size of the individual relative to the largest individual recorded in the region (calculated from a database of reef fish length estimates from the Northern Line Islands [18]). For example, an individual fish of length 30 cm for which the largest individual of that species was 40 cm would be assigned a value of 0.75. We computed station-specific mean estimates of length in units of standardized length (fraction of species-specific maximum length), and compared these estimates across islands for different trophic groups as described above.

To compare longevity across islands, we calculated the mean of the top quartile of age in years (T_max_, after [11]) for each species on each island, which allows us to compare across collections with small or variable sample sizes. We described growth using island-specific von Bertalanffy growth functions (VBGF): *L_t_* = *L_inf_* (1−exp([*−k*(*t*−*t_0_*)]), where *L_t_* is length at age *t*, *L_inf_* is mean asymptotic length, *k* describes the rate at which the asymptotic length is attained, *t* is the age in years, and *t_0_* is a theoretical age at which length is 0. Estimates of *t_0_* are sensitive to small sizes and ages; because our collections had few small/young individuals, we constrained *t_0_* to 0 in all cases [12]. We focus on the parameters *L_inf_* and *k*, which provide population-level estimates for mean maximum size (e.g. asymptotic length) and the rate at which that length is reached (a proxy for growth rate). However, since recent work has suggested modifications to the VBGF to enhance interpretability of the parameters *L_inf_* and *k* [40], we include calculations for a re-parameterized VBGF (Text S1, Table S1). To test whether growth curves differed significantly among atolls for each species, we compared AIC values for VBGF models that pooled samples between islands and VBGF models that fit growth curves separately for each island. For each species, statistical significance was determined in cases where the difference in AIC values (ΔAIC) between the 2-parameter and 4-parameter models was ≥2 [41]. We calculated 95% confidence regions around estimates of *L_inf_* and *k* following Kimura [42]. All analyses were conducted using R v.2.10 [43].

## Results

**Figure 2:**
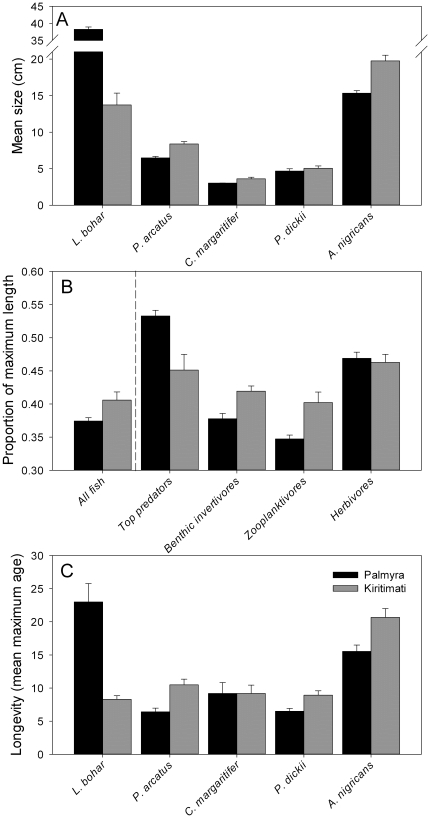
Size, maximum length and longevity between islands. A) Sizes of the five study species at each island from survey data (mean length ±1 SE). B) Proportion of maximum sizes for each trophic group and all fish combined from the full species assemblage for each island, based on maximum sizes for each species from survey data (mean proportion of maximum length ±1 SE). C) Longevity for the 5 study species (T_max_, mean of top quartile, ±95% CI). Data for Palmyra are in black, data for Kiritimati are in gray.

Abundance estimates differed between islands for all 5 species (p<0.05 in all cases; Table 1). Density of *L. bohar*, a top predator, was lower at fished Kiritimati relative to protected Palmyra. The remaining four prey species showed no consistent trend in abundance, with two species more abundant at Kiritimati (*C. margaritifer* and *P. dickii*) and two species more abundant at Palmyra (*P. arcatus* and *A. nigricans*), suggesting that numerical trophic cascades are not present in this system. However, despite the lack of evidence for classic trophic cascades, mean size as estimated by visual surveys differed significantly between islands for 4 of the 5 species (Fig. 2a). The mean size of *L. bohar* was larger at Palmyra relative to Kiritimati (p<0.05). In contrast, three of the four prey species, *P. arcatus*, *C. margaritifer*, and *A. nigricans*, were larger at Kiritimati (p<0.05; Fig. 2a). A similar relative pattern of size held across the entire fish assemblage. The mean size of top predators (estimated as a proportion relative to species-specific maximum sizes) was significantly larger at Palmyra. In contrast, the mean relative size of all fish was significantly larger at Kiritimati (Fig. 2b). Mean size of fish within two of the three trophic groups of prey, benthic invertivores and zooplanktivores, were significantly larger at Kiritimati (p<0.05), while herbivores did not differ in size between islands (Fig. 2b). Longevity, calculated as the mean of the top quartile of the collections [11], differed greatly between islands for 4 of the 5 species. Longevity for the predator *L. bohar* was significantly greater at Palmyra than Kiritimati, while longevity for three of the four prey species, *P. arcatus*, *P. dickii*, and *A. nigricans*, was significantly greater at Kiritimati versus Palmyra (p<0.001 in all cases; Fig. 2c). Longevity did not differ for *C. margaritifer* between islands.

**Figure 3:**
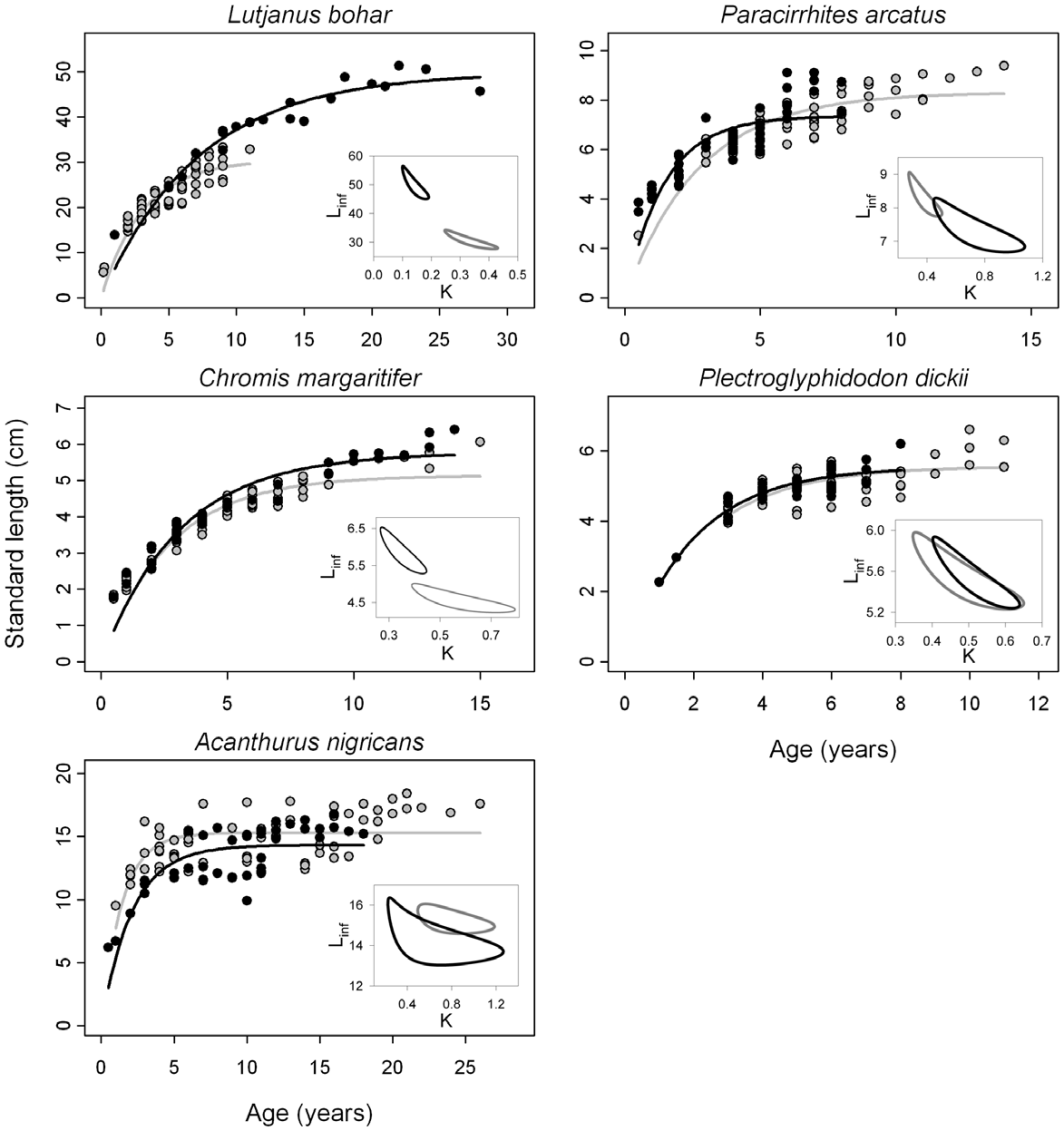
Size-at-age relationships for the 5 study species by island, with lines of best fit for VBGF parameters. Inset plots for each species show 95% confidence ellipses for the parameters *k* and *L_inf_*. Shading for histogram bars as in Fig. 2.

We estimated the parameters *L_inf_* and *k* from the VBGF for the five species from each island (Table 1). For each species, the parameters of the VBGF differed significantly between islands (ΔAIC≥2.0 for each species when comparing a 2-parameter model [i.e., pooling data between islands for each species] to a 4-parameter model [independent parameters fit for each island]). Inspection of 95% confidence intervals for the best-fit parameters of the species- and island-specific VBGFs illustrates the qualitative direction of difference for each species (Fig. 3). The estimate of *L_inf_* was significantly greater (and *k* was significantly smaller) at Palmyra relative to Kiritimati for the top predator, *L. bohar*, indicating that this species attained larger maximum sizes and reached those sizes at a slower rate at Palmyra compared to Kiritimati. A similar, but weaker, pattern existed for *C. margaritifer*. In contrast, *L_inf_* was significantly greater at Kiritimati relative to Palmyra for *P. arcatus* and *A. nigricans*, and *k* was significantly greater at Palmyra for *P. arcatus* (Fig. 3, Table 1). The estimate of *k* trended toward smaller values at Kiritimati relative to Palmyra for *P. dickii* (Fig. 3).

## Discussion

The Line Islands archipelago provides a rare opportunity to study the role played by top predator fishes in reef fish assemblages. Between fished Kiritimati (population of ∼10,000 with intensive subsistence fishing [44]) and unfished Palmyra (protected as a US National Wildlife Refuge since 2001), there is a >8-fold difference in biomass of top predators (and a >3-fold difference if reef sharks are excluded [18], [29]). In contrast to predators, there is no consistent pattern in the biomass or abundance of lower trophic levels [18], with no evidence of trophic cascades or even of simple prey release at the guild-level. Focusing on five of the most abundant species in the assemblage, we find a similar lack of clear density changes that are generally associated with tight trophic links. While the density of the top predator, *L. bohar*, was reduced at Kiritimati, the densities of the four prey species showed inconsistent patterns (i.e. two of the prey species had higher densities at Palmyra, and two had higher densities at Kiritimati; Table 1).

Even though we found no evidence for prey release and classic trophic cascades, changes in predator assemblages may still result in demographic changes on lower trophic levels that are more difficult to detect. We predicted that size structure would be shifted toward smaller sizes and longevity would be lower in prey fishes in locations where predator assemblages are intact. Furthermore, because of the trophic complexity of coral reef fish assemblages, we predicted that these demographic shifts would occur in the same direction for all lower trophic levels, and not alternate among trophic levels as in other systems as predicted by classical ecological theory [45], [46].

In general, differences in demographic parameters met our predictions for most species and trophic groups, particularly for mean size and longevity (T_max_, Fig. 2). Mean size differed as predicted for 3 out of 4 trophic groups and for 4 of 5 species, and longevity differed as predicted for 4 of 5 species (i.e., increases for the top predator and decreases for all lower trophic levels at unfished Palmyra). These results suggest that changes in predator assemblages can cause indirect demographic shifts throughout prey communities even where numerical prey release and trophic cascades are not apparent. While we predicted that predator-induced demographic shifts would include growth rate (here estimated by the parameter *k*), we found weak evidence for changes in growth rate. Only the top predator, *L. bohar* and the mid-level predator *P. arcatus* exhibited differences in the VBGF parameter *k*, with the apex predator growing slower and the mid-level predator growing faster at Palmyra, as predicted.

Estimates of maximum size (*L_inf_*) differed between islands for most species, in agreement with our predictions. The apex predator, *L. bohar* reached a significantly larger maximum size and maximum age at Palmyra, likely a result of the current ban on fishing in this U.S. National Wildlife Refuge and little extraction for decades previously. In contrast, species from lower trophic levels, including *P. arcatus*, *P. dickii*, and *A. nigricans* attained smaller maximum sizes and had reduced longevity at Palmyra, where overall top predator biomass is significantly greater [18], [29] and where predation pressure and/or predation risk is likely higher than at Kiritimati [47]. Our results complement the findings of other studies of life history changes in coral reef fishes in response to changes in predator abundance. Increases in apex predator biomass were correlated with decreases in size at sex change of many parrotfish species, when comparing the remote Northwestern Hawaiian Islands with the heavily fished main Hawaiian Islands [17] and when comparing four of the Northern Line Islands across a gradient of human disturbance [18]. Similar life history shifts in the size at sex change have been reported in Caribbean parrotfish in response to direct fishing pressure [7].

While fishing pressure is likely the strongest anthropogenic impact that varies between these islands, differences in habitat, coral disease, and microbial communities also exist. Coral cover is higher at Palmyra than Kiritimati, while coral disease and microbial concentrations are lower [29]. Loss of live coral and subsequent reduction in habitat complexity can lead to dramatic changes in reef fish communities [30], [48]–[50], including changes in top predator biomass. Reductions in live coral and habitat complexity on Kiritimati could further reduce abundance and size of top predators, thereby increasing differences in predator assemblages between islands and potentially increasing the magnitude of indirect effects on lower trophic levels. However, it is also likely that reductions of live coral cover and habitat complexity would negatively impact lower trophic groups dependent on those habitats and the shelter they provide [48], [49]. Still, our data cannot discount the possibility that additional anthropogenic stressors may have contributed to the patterns we observed.

Only *C. margaritifer* did not differ in T_max_ between islands, and our estimate of *L_inf_* was higher and *k* was lower for this species at Palmyra than at Kiritimati, counter to our predictions. There are a number of potential reasons why our predictions were unsupported for this species. First, as a planktivore, *C. margaritifer* may be dependent on pelagic delivery of zooplankton. The bathymetry is steeper and the currents often stronger at Palmyra than at Kiritimati [51], possibly increasing the rate of delivery of allochthonous zooplankton prey at Palmyra. Gut contents revealed that *C. margaritifer* had eaten more calanoid (planktonic) copepods compared to harpacticoid (benthic) copepods at Palmyra (E. Madin, *unpublished data*), indicating a greater reliance on pelagic prey. Changes in pelagic food supply could influence growth and longevity, and possibly counter the effects of increased predation pressure [13], [52]. Additionally, methodological concerns may have skewed results for *C. margaritifer*; for all other species, the relative difference in size across islands was concordant between the *in situ* count data (Fig. 2) and the collections (Fig. 3). In contrast, while count data suggest that *C. margaritifer* are larger at Kiritimati, the size distributions of our collections were indistinguishable (Palmyra: 3.99 cm; Kiritimati: 3.98 cm; p>0.95). Based on logistical constraints, our sampling methods for *C. margaritifer* (unlike the other four species) were quite different at the two islands: at Palmyra, we used BINCKE nets, which are more effective at capturing larger individuals of benthic fishes [53], while at Kiritimati we used clove oil, which is more effective at capturing smaller individuals. Therefore, our collections of *C. margaritifer* may not have been representative of populations at each island, which in turn may have influenced the demographic patterns we observed for this species.

A growing body of work has examined geographical variation in demography for a variety of fish species [11]. Many of these studies have examined demographic and life history variation of non-target species over large latitudinal scales, often attributing spatial variation across 100 s to 1000 s of km to differences in temperature [11], [12], [14], [15]. In contrast, Palmyra and Kiritimati are separated by less than 4° of latitude near the equator, and while Kiritimati experiences slightly more tropical upwelling than Palmyra, water temperatures generally differ by less than 1°C [29]. Increased tropical upwelling at Kiritimati also results in slightly higher productivity there. However, recent work has demonstrated that uninhabited, unfished equatorial atolls that experience as much or more tropical upwelling than Kiritimati have values of total fish biomass and top predator biomass that are similar to Palmyra and different from Kiritimati [29], [39]. Similar differences in regional demographic parameters have been found in areas with temperature differences of 5–8°C and up to 10-fold differences in productivity [13], [14], environmental differences that are far greater than those between Palmyra and Kiritimati. It is therefore most likely that the changes in demography we have observed between atolls in the Line Islands are the result of extensive inter-atoll differences in predator assemblages driven by human exploitation and not the result of environmental differences.

While differences in predator communities appear to have strong demographic consequences throughout the food web, the specific mechanisms by which longevity and size vary between islands are still uncertain. Increases in predator abundance may result in increased predation and direct increases in mortality of prey species, they may influence behavioral changes of prey species, or both. Behavioral changes may be related to prey species' assessment of predation risk while foraging, and can result in behaviorally mediated trophic cascades [54], [55]. Indeed, recent work suggests that the behavior of many of our study species changes between Palmyra and Kiritimati, such that prey spend more time sheltering, less time foraging, and exhibit more restricted movements at Palmyra where predators are more abundant than at Kiritimati [47]. Reductions in time spent foraging should decrease the total amount of resources consumed, which could in turn reduce the amount of energy available for growth and reproduction [13], [52]. Increases in predation risk may also alter life history tradeoffs between growth and reproduction, such that even less energy is allocated to growth and maintenance [56]. Additional evidence from these atolls suggest that species from lower trophic groups are less robust (i.e., weigh less at a given length) and have lower energy stores (i.e., relative liver size) at Palmyra where predators are more abundant [44], in accordance with life history theory. Our combined results suggest that predator-induced demographic shifts may be more pervasive than currently appreciated on coral reefs, and that the patterns we observed on unfished Palmyra may be closer to the state of these ecosystems before extensive human impacts.

## Supporting Information

Table S1 Reparameterized von Bertalanffy growth function parameter estimates for each species by island combination.
(DOC)

Text S1 Reparameterized von Bertalanffy growth function: methods and results.
(DOC)
