# Supplementary material for: Predator-Induced Demographic Shifts in Coral Reef Fish Assemblages
Source: PLoS One. 2011 Jun 16;6(6):e21062. doi: 10.1371/journal.pone.0021062 (PMC3116880; doi:10.1371/journal.pone.0021062)
Supplement: Table S1 — Reparameterized von Bertalanffy growth function parameter estimates for each species by island combination. (DOC) [file pone.0021062.s001.doc]

**Table S1.** Reparameterized von Bertalanffy growth function (rVBGF) parameter estimates (95% CI in parentheses)

|  |  | Parameter | | |
| --- | --- | --- | --- | --- |
| Species | Island | L(α) | L(β) | L(γ) |
| *L. bohar* | Kiritimati | 18.7 (18, 19.4) | 28.0 (26.9, 29.1) | 32.8 (29.8, 38.4) |
|  | Palmyra | 20.3 (13.9, 21.4) | 32.1 (30.7, 33.2) | 39.6 (38.4, 40.9) |
| *P. arcatus* | Kiritimati | 3.9 (3.4, 5.2) | 5.9 (5.7, 6.2) | 7.2 (6.9, 7.4) |
|  | Palmyra | 4.3 (4.2, 4.6) | 6.1 (5.8, 6.3) | 7.6 (7.2, 7.9) |
| *C. margaritifer* | Kiritimati | 2.2 (2.1, 2.3) | 3.6 (3.5, 3.6) | 4.5 (4.4, 4.5) |
|  | Palmyra | 2.3 (2.2, 2.5) | 3.7 (3.6, 3.8) | 4.7 (4.6, 4.8) |
| *P. dickii* | Kiritimati | 4.0 (1.2, 4.6) | 4.6 (4.4, 4.8) | 5.1 (4.9, 5.3) |
|  | Palmyra | 2.3 (2.1, 3.8) | 4.6 (4.4, 4.7) | 5.3 (5.1, 5.4) |
| *A. nigricans* | Kiritimati | 12.8 (9.4, 13.6) | 13.2 (12.4, 13.8) | 13.6 (13.1, 14.8) |
|  | Palmyra | 7.7 (7, 9.8) | 10.1 (9.4, 11) | 11.7 (10.9, 12.7) |
