# Supplementary material for: Predator-Induced Demographic Shifts in Coral Reef Fish Assemblages
Source: PLoS One. 2011 Jun 16;6(6):e21062. doi: 10.1371/journal.pone.0021062 (PMC3116880; doi:10.1371/journal.pone.0021062)
Supplement: Text S1 — Reparameterized von Bertalanffy growth function: methods and results. (DOC) [file pone.0021062.s002.doc]

**Supplemental text S1**

**Reparameterized von Bertalanffy growth function methods**

Recent work has raised questions about the interpretability of *L_inf_* and *k*, and a number of authors have proposed a re-parameterized version of the VBGF (e.g. Francis 1988). The re-parameterized version of the VBGF includes three parameters: L(α), L(β), L(γ), which estimate body size at arbitrary ages α, β, and γ. The ages α and γ are chosen to represent the early rapid growth phase and the later slower growth phase respectively, and β is the mean of α and γ. The re-parameterized version of the VBGF is:

$\text{L}_{\text{t}}\text{ }=\text{ L(}\alpha)+\frac{[L(\lambda)-L(\alpha)][1-r^{(2\frac{t-\alpha}{\lambda-\alpha})}]}{1-r^{2}}$, where L_t_ is length at time t, and $r=\frac{L(\lambda)-L(\beta)}{L(\beta)-L(\alpha)}$ subject to the constraints that L(α) < L(β) < L(γ) and [L(γ)- L(β)] ≠ [L(β) - L(α)].

Because a great deal of previous work examining age and growth in fishes used the traditional form of the VBGF but more recent work has shifted to the re-parameterized version of the VBGF (rVBGF), we report parameter estimates for the traditional VBGF in the body of the manuscript, and include parameter estimates for the rVBGF here to allow for comparisons across studies.

**Reparameterized von Bertalanffy growth function results**

For the rVBGF, we set α (age during early fast growth phase) to 1 year for all species, and set γ (adult age, when growth is slowing) to 6 years for *P. arcatus*, *C. margaritifer*, and *P. dickii*. We set γ to 12 years for *L. bohar* since it reaches adult size much later on Palmyra, and set γ to 5 years for *A. nigricans* because, despite its relatively long lifespan, it reaches asymptotic length relatively quickly, characteristic of many acanthurid species (Choat and Robertson 2002). For *L. bohar* and *C. margaritifer*, L(γ) and L(β) were significantly greater at Palmyra, while for *A. nigricans*, L(γ) and L(β) were significantly greater at Kiritimati, based on bootstrapped 95% confidence intervals. L(α), size at age 1, did not differ for any species between islands (Online Appendix Table 1).

**Appendix Literature Cited**

Choat, J. H., and D. R. Robertson. 2002. Age-based studies. Pages 57-80 *in* P. F. Sale, editor. Coral Reef Fishes: Dynamics and Diversity in a Complex Ecosystem. Academic Press, San Diego.

Francis, R. I. C. C. 1988. Are growth-parameters estimated from tagging and age length data comparable? Canadian Journal of Fisheries and Aquatic Sciences 45:936-942.
